# Paradigm Shift in Inflammatory Bowel Disease Management: Precision Medicine, Artificial Intelligence, and Emerging Therapies

**DOI:** 10.3390/jcm14051536

**Published:** 2025-02-25

**Authors:** Antonio M. Caballero Mateos, Guillermo A. Cañadas de la Fuente, Beatriz Gros

**Affiliations:** 1 Department of Internal Medicine, Gastroenterology Unit, Hospital Santa Ana, 18600 Motril, Spain; 2 Institute of Biosanitary Research (IBS) Precision Medicine, 18012 Granada, Spain; 3 Department of Nursing, Faculty of Health Sciences, University of Granada, 18016 Granada, Spain; gacf@ugr.es; 4 Brain, Mind and Behaviour Research Center (CIMCYC), University of Granada, Campus Universitario de Cartuja s/n, 18011 Granada, Spain; 5 Department of Gastroenterology and Hepatology, Reina Sofía University Hospital, IMIBIC, University of Cordoba, 14004 Cordoba, Spain; begrosal@gmail.com; 6 Biomedical Research Center in Hepatic and Digestive Disease, CIBEREHD, 28029 Madrid, Spain

**Keywords:** inflammatory bowel disease, Crohn’s disease, ulcerative colitis, precision medicine, artificial intelligence

## Abstract

Inflammatory bowel disease (IBD) management stands at the cusp of a transformative era, with recent breakthroughs heralding a paradigm shift in treatment strategies. Traditionally, IBD therapeutics revolved around immunosuppressants, but the landscape has evolved significantly. Recent approvals of etrasimod, upadacitinib, mirikizumab, and risankizumab have introduced novel mechanisms of action, offering renewed hope for IBD patients. These medications represent a departure from the status quo, breaking years of therapeutic stagnation. Precision medicine, involving Artificial Intelligence, is a pivotal aspect of this evolution, tailoring treatments based on genetic profiles, disease characteristics, and individual responses. This approach optimizes treatment efficacy, and paves the way for personalized care. Yet, the rising cost of IBD therapies, notably biologics, poses challenges, impacting healthcare budgets and patient access. Ongoing research strives to assess cost-effectiveness, guiding policy decisions to ensure equitable access to advanced treatments. Looking ahead, the future of IBD management holds great promise. Emerging therapies, precision medicine, and ongoing research into novel targets promise to reshape the IBD treatment landscape. As these advances continue to unfold, IBD patients can anticipate a brighter future, one marked by more effective, personalized, and accessible treatments.

## 1. Introduction

Crohn’s disease (CD) and Ulcerative Colitis (UC), both chronic inflammatory bowel diseases (IBDs), are conditions characterized by chronic relapsing-remittent inflammation of the gastrointestinal tract, significantly impacting patients’ quality of life. Despite the availability of conventional immunosuppressant-based therapies, managing IBDs remains a challenge due to limitations in their effectiveness and the potential for serious side effects [1]. This has sparked a critical need for novel treatment approaches that can effectively control the disease and improve patient outcomes.

In recent years, the landscape of IBD management has witnessed a promising paradigm shift, driven by the emergence of breakthrough therapies and the growing adoption of precision medicine principles. Precision medicine revolutionizes treatment by tailoring therapies to individual patient characteristics, such as genetic profiles, disease severity, and responses to previous therapies [2]. This personalized approach holds the promise of unlocking improved treatment efficacy and reducing adverse effects, providing hope for patients who have struggled with conventional therapies. Alongside precision medicine, the development of novel therapies with distinct mechanisms of action has further transformed IBD management [3,4]. Despite these advancements, the cost of IBD therapies remains a significant barrier, particularly for biologics, which is a reason why the biosimilar market has expanded in the last year. Ongoing research is dedicated to assessing the cost-effectiveness of these treatments and informing policy decisions to ensure equitable access for all patients [5]. This review aims to provide a comprehensive overview of emerging therapies, the role of precision medicine, and the integration of artificial intelligence in IBD management. By highlighting the most recent advancements, current limitations, and potential future directions, we seek to address gaps in knowledge regarding real-world applications, therapeutic challenges, and accessibility issues that impact patient care.

## 2. Novel Therapies and Emerging Targets

**Figure 1:**
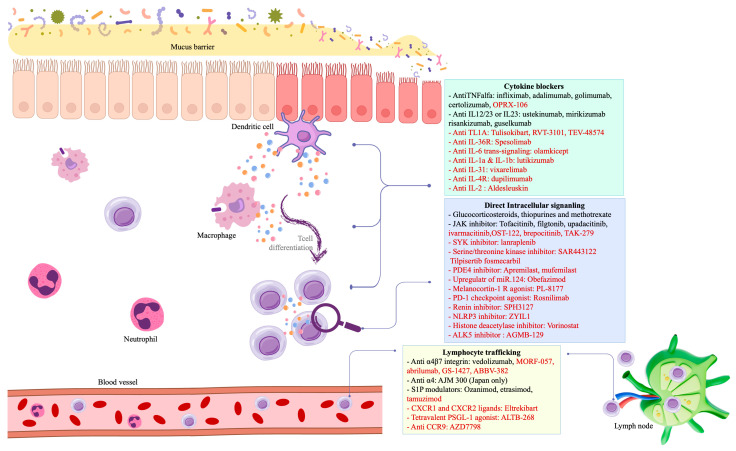
Interplay between mucosal immunology and pharmaceutical mechanisms in IBD. Drugs written in black are approved for IBD treatment, whereas those in red are currently in active phase 2/3 trials.

Significant strides have been made in the development of novel treatments for IBD, each targeting specific pathways implicated in the pathogenesis of involved in CD or UC [6]. The mechanism of action and clinical trials of promising novel therapies in CD and UC are summarized in Table 1 and Figure 1.

Interleukin (IL) inhibitors represent an important class of drugs that selectively target key cytokines involved in IBD inflammation [7]. For instance, risankizumab, guselkumab, and mirikizumab all aim to disrupt the IL-23 pathway [8,9,10]. IL-23 acts via JAK-STAT pathway promoting Th17 lymphocytes differentiation that induces further IL production, innate immune response, and leukocyte migration. Another emerging approach involves the selective inhibition of IL-36, a cytokine from the Il-1 family. IL-36 R signaling is thought to amplify the proliferation of gut cell populations that further promote recruitment and activate intestinal inflammation. Spesolimab, a humanized monoclonal antibody targeting the IL-36 receptor, works by blocking IL-36 signaling, thereby dampening the inflammatory response in UC and CD patients [11].

Selective inhibitors of IL-6 trans-signaling, such as olamkicept (sgp130Fc), offer another avenue for targeted therapy in IBD [12]. By specifically targeting IL-6 trans-signaling, these drugs disrupt the inflammatory cascade while minimizing potential side effects associated with global IL-6 blockade. Preliminary studies have demonstrated the clinical effectiveness of olamkicept in UC patients, paving the way for further investigation in larger clinical trials [13]. In addition, IL-33, a potent inflammatory cytokine, acting as both a pro-inflammatory factor and a transcriptional regulator, could be an interesting objective in future treatments due to its roles in innate and adaptive immunity [14]. Similarly, IL-1α and IL-1β, known pain mediators, play important roles in the pathogenesis of certain autoimmune diseases. Lutikizumab, an anti-IL-1 agent, is currently in phase 2 trials for UC and CD [15].

Therapies targeting TNF-alpha have long been cornerstone treatments for IBD. The TNF family includes a large number of cytokines, of which TNF-alpha is the best known. That being said, the latest advances have focused on tumor necrosis factor-like cytokine 1A (TL1A), another TNF molecule that is involved in the inflammatory cascade. Recent research has revealed that TL1A acts as a regulator mucosal immunity, and is involved in the immunological pathways that contribute to the development of IBD [16]. TL1A levels are elevated in the colonic mucosa of patients with UC and are associated with the severity of the disease. Thus, inhibiting TL1A could serve as a potential therapeutic target for treating inflammatory diseases, as seen in two preliminary studies in patients with UC [17,18]. Additionally, advancements are underway with emerging oral anti-TNF treatments like OPRX-106 and V565, promising new avenues for managing the condition [19,20]. These oral alternatives represent innovative approaches that could offer increased convenience and potentially enhanced effectiveness and treatment adherence for patients dealing with IBD. Several studies have corroborated a preference for oral treatments over other administration methods in patients with IBD. This has driven research efforts toward the development of effective oral therapies [21]. One such approach, currently in an experimental phase, involves the use of a robotic pill. The robotic pill is a novel oral device that navigates the gastrointestinal tract and releases its payload, such ustekinumab, directly to the small intestine [22]. It is nearly fully absorbable, minimizing the need for removal or other interventions. Additionally, in a more advanced stage, JNJ-77242113, a potent oral anti-IL-23 agent has been developed, which is showing promising efficacy in preliminary studies [23].

Beyond cytokine modulation, therapies targeting adhesion molecules have emerged as promising strategies for the management of IBD. Agents such as vedolizumab, currently approved for the treatment of both UC and CD, as well as investigational drugs like abrilumab, ontamalimab, and AJM-300, disrupt critical pathways involved in inflammation and immune cell trafficking, thereby modulating the disease process [24,25,26].

Looking ahead, the landscape of IBD management is poised for further transformation with the advent of sphingosine-1-phosphate receptor (S1PR) modulators, toll-like receptor (TLR) agonists, and microRNA-based therapies [27]. Drugs like ozanimod and etrasimod [28,29] are both currently approved for UC target S1PR receptors. By modulating S1PR signaling, these drugs have an anti-inflammatory function via sequestration of T cell subsets in the lymphoid tissues and prevention of gut homing in UC patients [30]. In addition, etrasimod recently demonstrated significant improvements versus placebo in patients with isolated proctitis [31].

JAK pathways have been demonstrated as crucial players in IBD [32]. Tofacitinib, filgotinib, and upadacitinib represent a significant advancement in IBD management, offering effective induction and maintenance of remission, especially for patients unresponsive to conventional therapies [33,34,35]. New generation JAK inhibitors, including izencitinib, ivarmacitinib, and peficitinib, have shown promise in clinical trials, mainly for UC [36,37,38].

Researchers are exploring various avenues for the treatment of IBD. Toll-like receptor 9 (TLR9) agonists might be of use in UC patients [39]. Cobitolimod is a synthetic single-stranded DNA molecule containing a CpG motif, a specific DNA sequence that TLR-9 recognizes as bacterial. By binding to TLR-9 on cells like intestinal Treg and B lymphocytes and antigen-presenting cells (APCs), cobitolimod triggers the release of potent anti-inflammatory cytokines, including IL-10 and type I interferons, helping to reduce inflammation [40]. Despite early promising results, the development of cobitolimod has been temporarily suspended, reflecting the complexities of advancing such treatments to broader clinical use. Similarly, phosphodiesterase (PDE) inhibitors, particularly PDE4 inhibitors like apremilast, have garnered attention for their potential to alleviate UC activity. An overexpression of PDE4 isoforms and a defective cAMP-mediated pathway were initially identified in active UC patients. Therapeutic inhibition of PDE4 via apremilast effectively modulated cAMP-dominant signaling through protein kinase A (PKA) and cAMP-response element-binding protein (CREB). This intervention led to clinical improvement in chronic UC, demonstrated by reduced mucosal ulcerations, decreased tissue fibrosis, and diminished inflammatory infiltration [41,42].

Recent studies have also focused on microRNAs (miRNAs) and their role in regulating gene expression in IBD. ABX464 (obefazimod) facilitates the targeted cutting and joining of a specific long non-coding RNA, resulting in the production of an anti-inflammatory microRNA known as miR-124. This new mechanism of action could help in inducing clinical remission in moderate-to-severe UC patients by reversing the expression of inflammatory cytokines [43].

Numerous phase 2 studies are investigating innovative treatments for UC, each with pioneering mechanisms of action. Lanraplenib, a spleen tyrosine kinase (SYK) inhibitor, is being tested for its ability to modulate immune cell signaling in B cells, monocytes, and macrophages, crucial cells in autoimmune disease pathways [44]. Additionally, receptor-interacting serine/threonine kinase inhibitors, such as tilpisertib fosmecarbil and eclitasertib (SAR443122), target RIPK1/2 pathways, which are central to immune responses through the nucleotide-binding oligomerization domain (NOD) and TLRs [45]. RIPK, expressed in antigen-presenting cells like dendritic cells and macrophages, responds to microbe-associated molecular patterns recognized by NOD1, NOD2, and TLRs. This interaction activates RIPK2, leading to the release of pro-inflammatory cytokines, including TNF-α, IL-6, and IL-12/23p40, which are central to the inflammatory response in UC. Another novel approach is rosnilimab, a PD-1 agonist antibody, designed to inhibit T-cell proliferation and cytokine secretion by depleting PD-1high T-cell subsets [46]. SPH3127, which targets the renin-angiotensin system, offers potential anti-inflammatory and antifibrotic effects, and is also under investigation [47]. Eltrekibart, a monoclonal antibody blocking CXCR1/2, aims to disrupt neutrophil extracellular trap formation, and is currently being tested in combination with mirikizumab [48]. Furthermore, leiolizumab (ALTB-268), a PSGL-1 agonist antibody, functions as an immune checkpoint enhancer to reduce T-cell effector activity, encouraging T-cell exhaustion [49]. Lastly, ZYIL1 targets the NLRP3 inflammasome, a complex that activates pro-inflammatory cytokines IL-1β and IL-18, aiming to control inflammation at the cellular level in UC [50]. For patients with mild to moderate UC who do not respond to 5-aminosalicylic acid (5-ASA), therapeutic options are scarce. MH002, a novel biotherapeutic product, consists of a carefully selected consortium of six non-pathogenic, commensal bacteria. These bacteria are well-characterized for their ability to modulate the immune response, promote tissue repair, and reinforce the integrity of the gut barrier, offering a potential new treatment pathway for UC, as seen in a recent randomized clinical trial [51].

New mechanisms of action are being investigated for treating CD, including immune system modulation by agents like vorinostat. Vorinostat, a histone deacetylase inhibitor (HDACi) with anti-cancer properties, has shown potential for regulating immune responses, though its precise mechanisms remain unclear. Studies have indicated that it reduces inflammation by inhibiting monocyte activation, T-cell immune responses, and dendritic cell functions, as well as suppressing Th1/Th17 cells and TNF-α levels, suggesting its utility in autoimmune diseases and conditions such as graft rejection [52]. Additionally, fibrosis-targeting therapies are gaining attention, particularly for fibrostenosing complications. AGMB-129, an oral GI-restricted small molecule inhibitor of ALK5 (TGFβR1), is designed to inhibit TGFβ, a key regulator of fibrosis, specifically within the GI tract [53].

Despite advances in medical therapy, surgery remains essential for refractory IBD or complications such as strictures, fistulas, and colorectal cancer. In CD, laparoscopic ileocecal resection is a viable alternative to anti-TNF therapy in selected cases, with lower recurrence rates [54]. Early bowel resection has also been associated with reduced long-term recurrence and a lower need for postoperative biologics. In UC, total proctocolectomy with ileal pouch–anal anastomosis (IPAA) remains as the preferred surgical approach for medically refractory patients [55]. Minimally invasive techniques, including stricturoplasty and endoscopic balloon dilation, provide alternatives to resection in Crohn’s-related strictures [56]. Optimized perioperative care and multidisciplinary management have significantly improved surgical outcomes in IBD [57].

The evolving landscape of IBD treatment is marked by promising advancements across various therapeutic avenues. Additionally, while challenges persist, such as meeting primary endpoints consistently in clinical trials, the ongoing pursuit of novel treatments underscores the dedication to improving outcomes for patients with IBD. As research progresses and new insights emerge, the hope is to continue refining therapeutic strategies, ultimately enhancing the quality of life for individuals living with these chronic inflammatory conditions.

## 3. The Rise of Precision Medicine in IBD Management

Precision medicine, a rapidly evolving approach to healthcare, aims to tailor treatment to individual patient characteristics, unlocking personalized treatment strategies that optimize efficacy and minimize adverse effects. This personalized approach will very likely transform IBD management [58]. Despite advancements in molecular biology and omics technologies (genomics, proteomics, metagenomics, and metabolomics), understanding IBD’s complexity remains difficult, largely due to the heterogeneity of the data and the lack of standardized analytical pipelines [59].

Artificial intelligence (AI) has emerged as an outstanding tool to address some of these challenges. IBD entails great complexity before diagnosis, and this becomes even more complex once the disease is diagnosed, due to the relapsing course, progressiveness, and lack of response, among other scenarios. Of particular interest is AI’s role in identifying non-invasive biomarkers. AI has been applied to various omics studies in IBD, including genomics, transcriptomics, and microbiomics, with the goal of improving diagnosis, predicting therapeutic response, and understanding disease progression [60]. In genomic studies, AI models leveraging genome-wide association studies’ (GWASs) data have demonstrated better performance than whole-exome sequencing (WES) in distinguishing CD from UC, likely due to the larger sample sizes available in GWASs [61]. Transcriptomic studies using gene expression data from microarrays and RNA sequencing have been effective in differentiating between UC, healthy controls, and other diseases [62,63]. These advancements highlight AI’s potential utility in the diagnostic phases of IBD management by improving accuracy and enabling earlier disease classification.

Genetic testing is being utilized to identify patients with certain genetic variants associated with severe IBD, who may benefit from more intensive therapies [64]. For example, AI models based on single-cell RNA-seq data have been applied to identify inflammatory phenotypes and predict responses to biologic therapies like vedolizumab [65]. Additionally, personalized biologics, designed to selectively target specific immune pathways, are demonstrating remarkable efficacy in treating subsets of patients with IBD [66]. However, in fields like proteomics, AI applications are less advanced, and most studies still rely on traditional statistical approaches.

In the study of the intestinal microbiota, AI has been used to integrate microbiomic data with clinical and demographic parameters, showing potential for predicting disease progression and therapy response [67]. AI is also making strides in histological and endoscopic evaluations, which are crucial for IBD diagnosis and monitoring. AI can accurately identify microscopic disease features, predict histological remission, anticipate flare-ups, and optimize therapeutic management. AI-enhanced endoscopy could improve the detection of subtle mucosal changes, aiding diagnosis, real-time disease activity assessment, and evaluation in clinical trials. However, there remain challenges in developing AI tools that can be broadly applied due to selection bias and data variability [68,69]. In the coming years, international data-sharing initiatives will be key to training AI on comprehensive, unbiased datasets that better reflect the diversity of IBD patient populations.

One of the most validated applications of AI in IBD management is in endoscopic evaluation. AI-assisted systems such as EndoBrain^®^ and CAD-EYE^®^ have been developed to enhance the detection of dysplasia and inflammatory lesions in patients with UC, improving diagnostic accuracy and reducing interobserver variability [70]. Additionally, convolutional neural networks (CNNs) have been trained to identify endoscopic disease activity with accuracy comparable to expert gastroenterologists. Recent studies demonstrate that AI models can automate the classification of endoscopic severity using standardized scoring systems, including the Mayo Endoscopic Score and the Ulcerative Colitis Endoscopic Index of Severity (UCEIS), offering an objective and reproducible assessment of disease progression [71,72].

Another emerging application of AI in IBD management is the use of generative AI models, such as ChatGPT and other large language models (LLMs) (Gemini, LLaMA, Deepseek…), to assist patients and healthcare professionals. These AI-driven tools have been explored for patient education, symptom tracking, and personalized treatment guidance. Chatbots powered by generative AI can provide 24/7 support to patients, answering questions about medications, dietary recommendations, and disease management strategies, improving adherence to treatment and reducing the burden on healthcare providers [73]. Moreover, AI-assisted clinical decision support systems are being developed to integrate patient-reported symptoms, laboratory data, and imaging findings to optimize treatment adjustments in real time [74].

As these tools evolve, standardized AI-based methods are expected to improve histopathology workflows, enabling more precise differentiation between IBD subtypes and between IBD and non-IBD conditions. The integration of multi-omics data with AI offers tremendous potential, but real-world clinical applications are still emerging. Nevertheless, AI’s future role in IBD management looks promising, with the potential to enhance personalized treatment strategies, predict disease progression, and refine diagnostic accuracy. Precision medicine, along with advancements in AI and other novel therapies, offers the potential to improve the quality of life for IBD patients, aiming to reduce the disease’s impact and better manage symptoms.

## 4. Navigating the Financial Landscape of IBD Management

In the landscape of IBD management, the complexities extend beyond medical intricacies to financial challenges. Despite significant strides in therapeutic options, including the advent of biologics, the burden of managing IBD remains substantial, primarily due to the rising costs associated with treatment modalities [75,76]. This conundrum presents a multifaceted dilemma for healthcare systems, providers, and, most importantly, patients.

At the forefront of the cost challenge are biologics, which have redefined the treatment paradigm for IBD. While these therapies offer unprecedented efficacy and the promise of sustained remission, their high prices often place them beyond the reach of many patients and strain healthcare budgets. Factors contributing to the high costs of biologics include complex manufacturing processes, stringent intellectual property protections, and the significant demand for these life-changing treatments [77]. Consequently, patients with limited financial means face formidable barriers in accessing these potentially life-saving therapies, exacerbating health disparities and compromising treatment outcomes. The cost of IBD care continues to rise, driven largely by the increasing use of biologics and small-molecule therapies, which now account for 75% of total CD and 50% of UC costs within five years of diagnosis [78]. While these treatments have significantly improved patient outcomes, their high cost poses challenges for healthcare sustainability, particularly in regions with limited drug price regulation.

Biosimilars present a viable solution, offering comparable efficacy at significantly reduced prices [79]. Denmark achieved an 83% cost reduction in adalimumab expenditures within three months of biosimilar adoption [80], while Canada reported 20–50% savings in drug expenses. Cost-effectiveness studies show biosimilars can reduce treatment costs by 30–50% compared to originator biologics, increasing accessibility without compromising clinical outcomes [81]. However, regulatory barriers and reimbursement policies in some regions continue to slow their widespread adoption. Looking ahead, the introduction of new biosimilars, such as ustekinumab biosimilars and tofacitinib generics, alongside emerging treatments, is anticipated to influence cost reduction in IBD management. By embracing these innovations and implementing cost-effective strategies, healthcare systems could better meet the needs of patients with IBD while ensuring sustainable resource allocation. However, successful integration of biosimilars into clinical practice necessitates robust regulatory frameworks, clinician education, and patient engagement initiatives to foster confidence and acceptance [82].

To enhance cost sustainability, early treat-to-target strategies, telemedicine, and policy reforms are essential. Multidisciplinary care models, the introduction of AI, and remote patient monitoring have shown cost savings by reducing hospitalizations and emergency visits, while price negotiations and biosimilar incentives could further optimize healthcare spending [83]. As treatment advances, a balance between innovation and affordability is key to ensuring equitable access to effective IBD therapies. By fostering collaboration among stakeholders, investing in research and development, and embracing progressive policies, we can pave the way for a future where all individuals affected by IBD have equitable access to the full spectrum of treatment options, irrespective of their socioeconomic status [5]. Through collective action and unwavering commitment, we can navigate the financial landscape of IBD management with compassion, integrity, and innovation, ensuring that no patient is left behind in their journey towards health and wellness.

## 5. Ensuring Safety in IBD Treatment

As the landscape of IBD management continues to evolve, concerted efforts are essential to strike a delicate balance between advancing therapeutic innovation and ensuring affordability, accessibility and safety. Clinical trials recruitment is becoming more and more complex for a number of reasons, among which placebo use has been a concerning one [84]. In addition, understanding the safety profiles of biologics and small molecules is essential for treatment selection. While anti-TNF agents remain widely used, they are associated with serious infections, malignancies (e.g., melanoma, lymphoma), and immunogenicity, which can reduce their efficacy [85]. In contrast, anti-integrins (vedolizumab) and anti-interleukins (ustekinumab, risankizumab) have a more favorable safety profile, though risks such as respiratory infections should be considered [86]. Recently approved small molecules, including JAK inhibitors (tofacitinib, upadacitinib) and S1PR modulators (ozanimod), provide oral alternatives but come with unique risks, such as cardiovascular events, venous thromboembolism, and serious infections (e.g., herpes zoster reactivation). Treatment selection must be guided by patient-specific factors, such as malignancy history and cardiovascular risk, alongside regular safety monitoring protocols to mitigate adverse effects [87].

Future therapies in IBD are under investigation, targeting new inflammatory pathways beyond current treatments. While promising for refractory cases, their long-term safety remains unknown, requiring rigorous clinical trials and post-marketing surveillance. A risk-stratification approach will be essential to integrate these therapies safely, balancing efficacy and risk in the expanding IBD treatment landscape.

## 6. Limitations and Future Directions

This review provides an updated perspective on key advances and future directions in IBD management, covering novel therapies, precision medicine, artificial intelligence applications, and healthcare cost challenges. However, several limitations must be acknowledged. Firstly, this is a non-systematic review, meaning it does not follow a structured methodology for literature selection and synthesis. While this approach allows for a broad discussion of cutting-edge aspects, it does not provide a comprehensive analysis of all available evidence, which systematic reviews typically offer. Secondly, although precision medicine and artificial intelligence hold great promise for optimizing IBD management, their real-world implementation remains complex. Genetic variability, disease heterogeneity, and the need for standardization in omics data analysis and AI-driven models present ongoing challenges. Further research is required to ensure the reproducibility and clinical applicability of these technologies.

Additionally, one notable limitation of this review is the lack of discussion on pediatric IBD. Pediatric IBD often presents with more extensive disease at diagnosis, compared to adults. Growth failure and delayed puberty are major concerns in this population, which were not addressed in our study. Pediatric-onset IBD accounts for 20–30% of diagnoses, and is often more aggressive than adult-onset disease. CD is more common than UC, and pediatric UC frequently presents as extensive pancolitis [88]. Additionally, children have a higher risk of surgery within five years of diagnosis. Furthermore, pediatric IBD has a stronger genetic component, and may require different treatment strategies due to distinct disease progression patterns and long-term safety considerations. Very early-onset IBD is more likely to have a monogenic origin, affecting immune regulation and barrier function [89]. Biologics are increasingly used early, but data on newer therapies in children are limited. Despite advances, surgical rates remain high, often due to growth failure or refractory disease, requiring tailored surgical approaches. Future research should focus on age-specific treatment approaches and long-term outcomes in pediatric IBD, bridging the gap between pediatric and adult management strategies.

The future of IBD management is set to be transformed by advancements in precision medicine, AI, and innovative therapeutic strategies. Looking forward, research should prioritize identifying specific patient subgroups that will benefit most from targeted therapies, optimizing precision medicine approaches [90]. Chimeric Antigen Receptor T-cell Therapy (CAR-T) cell therapy is gaining interest in the treatment of autoimmune diseases, including IBD. CAR-T cell therapy involves genetically engineering a patient’s own T cells to target and eliminate specific immune cells responsible for the autoimmune response. The potential benefits of CAR-T cell therapy include the ability to specifically target and eliminate the autoreactive immune cells responsible for the disease, hypothetically leading to a better management of the disease [91]. This approach may also avoid the need for long-term immunosuppressive therapy, which are not free of side effects [92]. Additionally, microbiome-based therapies, including next-generation probiotics and fecal microbiota transplantation (FMT), are emerging as promising strategies to restore gut homeostasis and improve treatment efficacy [93]. Beyond inflammation control, future therapies will likely target fibrosis modulation, epithelial barrier repair, and the gut–brain axis, addressing complications like strictures and motility disorders [94,95]. The rise of digital health tools, such as wearable biosensors and AI-powered symptom-tracking apps, will enable real-time monitoring and personalized disease management, improving patient adherence and outcomes [96]. As research advances, a multidisciplinary approach integrating precision medicine, novel therapeutics, and digital innovations will reshape the IBD treatment paradigm, offering more effective, personalized, and accessible care.

## 7. Conclusions

Recent advancements in CD and UC management offer new hope for patients. Precision medicine, AI, and novel therapies targeting specific inflammatory pathways show promise in improving treatment efficacy and minimizing adverse effects. However, the rising costs of biologics present challenges in access and affordability. The introduction of biosimilars and emerging treatments may alleviate this burden, provided robust regulatory frameworks and clinician education are in place. AI-driven tools have the potential to revolutionize IBD management by enhancing diagnostic accuracy, predicting disease progression, and personalizing treatment strategies. The integration of multi-omics data with AI is particularly promising in uncovering disease mechanisms and identifying non-invasive biomarkers, further advancing precision medicine.

Moving forward, collaborative efforts, investment in research, and progressive policies are crucial to ensuring equitable access to effective and affordable IBD treatments. By prioritizing patient-centered care and addressing socioeconomic disparities, we can strive towards a future where all individuals affected by IBD receive optimal care.

## Figures and Tables

**Table 1:** Comprehensive review of approved advanced molecules and active phase 2/3 trials in IBD.

Drug	Mechanism of Action	Indication	Phase or Approval	Identification Number
**Infliximab**	**Anti-TNFα**	**CD and UC**	**Approved by EMA and FDA**	
**Adalimumab**	**Anti-TNFα**	**CD and UC**	**Approved by EMA and FDA**	
**Certolizumab**	**Anti-TNFα**	**CD**	**Approved by FDA**	
**Golimumab**	**Anti-TNFα**	**UC**	**Approved by EMA and FDA**	
**Vedolizumab**	**Anti-α4β7 Integrin**	**CD and UC**	**Approved by EMA and FDA**	
**Ustekinumab**	**Anti-IL-12/23**	**CD and UC**	**Approved by EMA and FDA**	
**Risankizumab**	**Anti-IL-23**	**CD and UC**	**Approved by EMA and FDA**	
**Guselkumab**	**Anti-IL-23**	**UC** CD	**Approved by FDA** Ongoing phase 3 (GALAXI)	NCT05347095 NCT06408935
**Mirikizumab**	**Anti-IL-23**	**UC** CD	**Approved by EMA and FDA** Published phase 3 awaiting approval	NCT03926130
**Tofacitinib**	**JAK Inhibitor**	**UC**	**Approved by EMA and FDA**	
**Filgotinib**	**JAK Inhibitor**	**UC**	**Approved by EMA**	
**Upadacitinib**	**JAK Inhibitor**	**CD and UC**	**Approved by EMA and FDA**	
**Ozanimod**	**S1P Modulator**	**UC** CD	**Approved by EMA and FDA** Ongoing Phase 3	NCT03440385 NCT03440372
**Etrasimod**	**S1P Modulator**	**UC** CD	**Approved by EMA and FDA** Ongoing Phase 3	NCT04173273
Tamuzimod (VTX 0002)	S1P Modulator	UC	Terminated Phase 2	NCT05156125
Spesolimab (BI 655130)	Anti IL-36R	UC CD	Ongoing Phase 2 Ongoing Phase 2	NCT03482635 NCT03752970
Olamkicept (TJ301)	Anti IL-6 trans-signaling	UC	Completed Phase 2 No phase 3 trial registered	NCT03235752
Apremilast	PDE4 inhibitor	UC	Completed Phase 2 No phase 3 trial registered	NCT02289417
Obefazimod (ABX464)	Upregulator of miR-124	UC	Ongoing Phase 3	NCT05507203 NCT05507216
PL-8177	Melanocortin-1 receptor agonist	UC	Ongoing Phase 2	NCT05466890
Lutikizumab	Anti IL-1a and IL-1b	UC CD	Ongoing Phase 2 Ongoing Phase 2	NCT06257875 NCT06548542
Vixarelimab	Anti IL-31 and oncostatin M	UC	Ongoing Phase 2	NCT06137183
Lanraplenib (BI 3032950)	Inhibition of spleen tyrosine kinase (SYK)	UC	Ongoing Phase 2	NCT06636656
Tilpisertib fosmecarbil (GS-5290)	Serine/threonine kinase inhibitor	UC	Ongoing Phase 2	NCT06029972
SAR443122	serine/threonine protein kinase 1	UC	Ongoing Phase 2	NCT05588843
Rosnilimab	PD-1 checkpoint agonist	UC	Ongoing Phase 2	NCT06127043
SPH3127	Renin inhibitor	UC	Ongoing Phase 2	NCT05019742 NCT05770609
Eltrekibart (DB19017)	CXCR1 and CXCR2 ligands	UC	Ongoing Phase 2 in association with mirikizumab	NCT06598943
ALTB-268	Tetravalent PSGL-1 agonist antibody	UC	Ongoing Phase 2	NCT06109441
ZYIL1	NLRP3 inflammasome inhibitor	UC	Ongoing Phase 2	NCT06398808
Dupilimumab	Anti IL-4R	UC	Ongoing Phase 2	NCT05731128
Mufemilast (Hemay005)	PDE4 inhibitor	UC	Ongoing Phase 2	NCT05486104
Vorinostat	Histone deacetylase inhibitor	CD	Ongoing phase 1/2(in combination with ustekinumab)	NCT03167437
Aldesleuskin	IL-2 inhibitor	CD	Ongoing Phase 1/2	NCT04263831
AGMB-129	ALK5 inhibitor	Fibrostenotic CD	Ongoing Phase 2	NCT05843578
AZD7798	Anti CCR9	CD	Ongoing Phase 2	NCT06450197
Abrilumab	Anti α4β7 integrin	UC	Completed Phase 2 No phase 3 trial registered	NCT01694485
GS-1427	Anti α4β7 integrin	UC	Ongoing Phase 2	NCT06290934
AJM-300	Anti α4	UC	Completed Phase 3	NCT03531892
ABBV-382	Anti α4β7 integrin	CD	Ongoing Phase 2	NCT06548542
MORF-057	Oral anti α4β7	UC CD	Ongoing Phase 2 Ongoing Phase 2	NCT05611671 NCT06226883
Ontamalimab (PF-00547659)	Anti-MAdCAM-1	CD and UC	Completed Phase 3	NCT03259334 NCT03259308 NCT03290781 NCT03559517 NCT03566823 NCT03627091
Tamuzimod	S1P modulator	UC	Ongoing Phase 2	NCT05156125
Ivarmacitinib	JAK inhibitor	CD UC	Completed Phase 2 Ongoing Phase 3	NCT03677648 NCT05181137
Brepocitinib	JAK inhibitor	UC	Completed Phase 2 No Phase 3 trial registered	NCT02958865
OST-122	JAK3/TYK2/ARK5	UC	Completed Phase 1b/2a	NCT04353791
Zasocitinib (previously TAK-279)	TYK2 inhibitor	UC CD	Ongoing Phase 2 Ongoing Phase 2	NCT06254950 NCT06233461
OPRX-106	Anti TNF	UC	Completed Phase 2 No Phase 3 trial registered	NCT02768974
RVT-3101 (previously PF-06480605)	Anti TL1A	UC	Ongoing phase 2b	NCT04090411
Tulisokibart (MK-7240, previously known as PRA-023)	Anti TL1A	UC CD	Ongoing Phase 3 Ongoing Phase 3	NCT06052059 NCT06651281 NCT06430801
TEV-48574	Anti TL1A	UC CD	Ongoing Phase 2 Ongoing Phase 2	NCT05668013 NCT05499130

Note: The data presented in this table were extracted from ClinicalTrials.gov by filtering for all currently active clinical trials investigating advanced new drugs for inflammatory bowel disease. The search included phase 2 and phase 3 trials, and was performed using relevant keywords and condition filters. Only interventional studies with ongoing recruitment or active status were considered. Approved drugs are shown in bold. The information was retrieved and summarized as of 3 December 2024.

## Abbreviations

IBD	Inflammatory Bowel Disease
CD	Crohn’s Disease
UC	Ulcerative Colitis
TNF	Tumor Necrosis Factor
IL	Interleukin
JAK	Janus Kinase
S1P	Sphingosine-1-Phosphate
PDE	Phosphodiesterase
SYK	Spleen Tyrosine Kinase
TLR	Toll-Like Receptor
miRNA	MicroRNA
GWAS	Genome-Wide Association Studies
WES	Whole-Exome Sequencing
AI	Artificial Intelligence
CNN	Convolutional Neural Network
UCEIS	Ulcerative Colitis Endoscopic Index of Severity
EMA	European Medicines Agency
FDA	Food and Drug Administration
MAdCAM-1	Mucosal Addressin Cell Adhesion Molecule-1
CAR-T	Chimeric Antigen Receptor T-cell Therapy
PDE4	Phosphodiesterase-4
ALK5	Activin-like Kinase 5
PSGL-1	P-Selectin Glycoprotein Ligand-1
NLRP3	NOD-, LRR-, and pyrin domain-containing protein 3
RIPK1/2	Receptor-Interacting Protein Kinase 1/2
CXCR1/2	C-X-C Motif Chemokine Receptor 1/2
HDACi	Histone Deacetylase Inhibitor
CCR9	C-C Motif Chemokine Receptor 9
TYK2	Tyrosine Kinase 2
PD-1	Programmed Death-1
IPAA	Ileal Pouch-Anal Anastomosis
cAMP	Cyclic Adenosine Monophosphate
CREB	cAMP Response Element-Binding Protein
Treg	Regulatory T Cells
APC	Antigen-Presenting Cell
